# 2-(1-Adamant­yl)-1-(3-amino­phen­yl)ethanol

**DOI:** 10.1107/S1600536811034763

**Published:** 2011-08-27

**Authors:** Michal Rouchal, Zuzana Kozubková, Marek Nečas, Robert Vícha

**Affiliations:** a Department of Chemistry, Faculty of Technology, Tomas Bata University in Zlin, Nám. T. G. Masaryka 275, Zlín,762 72, Czech Republic; b Department of Chemistry, Faculty of Science, Masaryk University, Kamenice 5, Brno-Bohunice, 625 00, Czech Republic

## Abstract

In the crystal structure of the title compound, C_18_H_25_NO, mol­ecules are linked *via* O—H⋯N hydrogen bonds, forming chains parallel to the *c* axis. Additional weak N—H⋯O inter­actions stabilize the crystal packing. The adamantane cage consists of three fused cyclo­hexane rings in almost ideal chair conformations, with C—C—C angles in the range 107.9 (10)–111.3 (11)°.

## Related literature

For the biological activity of adamantane-bearing compounds, see: van der Schyf & Geldenhuys (2009) ▶. For related structures, see: Rouchal *et al.* (2009 ▶, 2010 ▶).
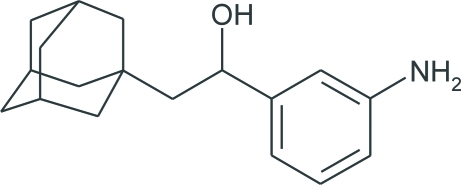


## Experimental

### 

#### Crystal data

C_18_H_25_NO
*M* *_r_* = 271.39
Orthorhombic, 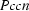

*a* = 16.4467 (7) Å
*b* = 22.1873 (9) Å
*c* = 8.1033 (4) Å
*V* = 2957.0 (2) Å^3^
*Z* = 8
Mo *K*α radiation
μ = 0.07 mm^−1^
*T* = 120 K
0.30 × 0.20 × 0.10 mm

#### Data collection

Kuma KM-4 CCD diffractometer
Absorption correction: multi-scan (*CrysAlis RED*; Oxford Diffraction, 2009 ▶) *T*_min_ = 0.984, *T*_max_ = 1.000
30937 measured reflections
2602 independent reflections
1716 reflections with *I* > 2σ(*I*)
*R*_int_ = 0.053

#### Refinement

*R*[*F* ^2^ > 2σ(*F* ^2^)] = 0.032
*wR*(*F* ^2^) = 0.068
*S* = 0.85
2602 reflections
190 parameters
H atoms treated by a mixture of independent and constrained refinement
Δρ_max_ = 0.14 e Å^−3^
Δρ_min_ = −0.15 e Å^−3^

### 

Data collection: *CrysAlis CCD* (Oxford Diffraction, 2009 ▶); cell refinement: *CrysAlis RED* (Oxford Diffraction, 2009 ▶); data reduction: *CrysAlis RED*; program(s) used to solve structure: *SHELXS97* (Sheldrick, 2008 ▶); program(s) used to refine structure: *SHELXL97* (Sheldrick, 2008 ▶); molecular graphics: *ORTEP-3* (Farrugia, 1997 ▶) and *Mercury* (Macrae *et al.*, 2008 ▶); software used to prepare material for publication: *SHELXL97*.

## Supplementary Material

Crystal structure: contains datablock(s) global, I. DOI: 10.1107/S1600536811034763/pk2344sup1.cif

Structure factors: contains datablock(s) I. DOI: 10.1107/S1600536811034763/pk2344Isup2.hkl

Supplementary material file. DOI: 10.1107/S1600536811034763/pk2344Isup3.cml

Additional supplementary materials:  crystallographic information; 3D view; checkCIF report

## Figures and Tables

**Table 1:** Hydrogen-bond geometry (Å, °)

*D*—H⋯*A*	*D*—H	H⋯*A*	*D*⋯*A*	*D*—H⋯*A*
O1—H1*A*⋯N1^i^	0.84	2.10	2.9400 (14)	176
N1—H1*C*⋯O1^ii^	0.930 (15)	2.295 (15)	3.2048 (16)	166.0 (13)
N1—H1*B*⋯O1^iii^	0.930 (16)	2.357 (16)	3.2472 (16)	160.1 (14)

Symmetry codes: (i) 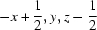; (ii) 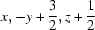; (iii) 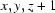.

**Fig. 1.**
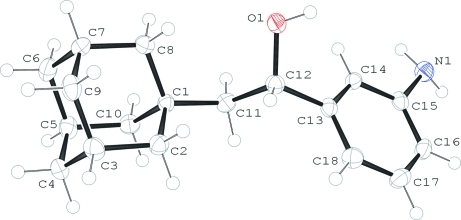
Ellipsoid plot of the asymmetric unit with atoms represented as 50% probability ellipsoids. Hydrogen atoms are shown as small spheres of arbitrary radius.

**Fig. 2.**
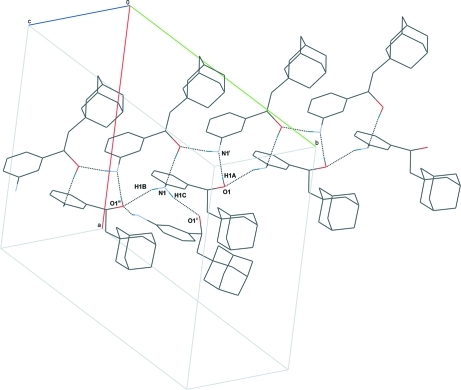
Part of the crystal structure of the title compound showing the H-bonds (dashed lines). H-atoms (except those which are involved in H-bonding) have been omitted for clarity. Symmetry codes: (i) -x+0.5,y,z-0.5; (ii) x,-y+1.5,z+0.5; (iii) x,y,z+1.

## supplementary crystallographic information

### Comment

It is matter of common knowledge that the well advised introduction of the highly lipophilic adamantane moiety into biologically active compounds might improve some pharmacological properties of the resulting molecule (van der Schyf & Geldenhuys, 2009). The title compound belongs to the series of recently synthesized building blocks for drug modification based on adamantylated aromatic amines.

The asymmetric unit of the title compound consists of a single molecule (Fig. 1). The benzene ring is nearly planar with a maximum deviation from the best plane being 0.006 (13) Å for C13. The torsion angles describing an arrangement of adamantane cage, benzene ring and aliphatic linker C1–C11–C12–C13, C11–C12–C13–C18, and C10–C1–C11–C12 are 158.37 (11), -95.75 (14), and -178.42 (11)°, respectively. The presented structure is linked into pairs by O–H···N hydrogen bonds (Fig. 2, Table 1). The crystal packing is further stabilized *via* intermolecular N–H···O interactions (Table 1).

### Experimental

2-(1-Adamantyl)-1-(3-nitrophenyl)ethanol (350 mg, 1.16 mmol) was dissolved in methanol (34 cm^3^) and 7 cm^3^ of hydrochloric acid/water (1/1, *v*/*v*) was added. Into the refluxed and well stirred mixture, portions of an iron powder were added successively. The reaction was stopped when TLC indicated the consumption of all starting material. The mixture was neutralized with 5% solution of NaOH (50 cm^3^) and extracted with diethyl ether (6 × 10 cm^3^). Combined organic layers were twice washed with brine, dried over sodium sulfate and evaporated in vacuum. The purification of crude material by washing with hexane provided the desired product as a colourless crystalline powder (258 mg, 82%, mp 415–418 K). The crystal used for data collection was grown by spontaneous evaporation from diethyl ether at room temperature.

### Refinement

All carbon bound H atoms were placed at calculated positions and were refined as riding with their *U*_iso_ set to 1.2*U*_eq_ of the respective carrier atoms. The oxygen bound hydrogen was placed at calculated coordinates refined with a torsional degree of freedom, and with *U*_iso_ set to 1.5*U*_eq_ of the carrier atom. Nitrogen bound H atoms were located in a difference Fourier map and refined isotropically.

### Crystal data

**Table:**

C_18_H_25_NO	*D*_x_ = 1.219 Mg m^−^^3^
*M**_r_* = 271.39	Melting point: 417 K
Orthorhombic, *P**c**c**n*	Mo *K*α radiation, λ = 0.71073 Å
Hall symbol: -P 2ab 2ac	Cell parameters from 6715 reflections
*a* = 16.4467 (7) Å	θ = 2.9–27.3°
*b* = 22.1873 (9) Å	µ = 0.07 mm^−^^1^
*c* = 8.1033 (4) Å	*T* = 120 K
*V* = 2957.0 (2) Å^3^	Block, colourless
*Z* = 8	0.30 × 0.20 × 0.10 mm
*F*(000) = 1184	

### Data collection

**Table:**

Kuma KM-4 CCD diffractometer	2602 independent reflections
Radiation source: fine-focus sealed tube	1716 reflections with *I* > 2σ(*I*)
graphite	*R*_int_ = 0.053
Detector resolution: 0.06 pixels mm^-1^	θ_max_ = 25.0°, θ_min_ = 3.0°
ω scans	*h* = −18→19
Absorption correction: multi-scan (*CrysAlis RED*; Oxford Diffraction, 2009)	*k* = −26→26
*T*_min_ = 0.984, *T*_max_ = 1.000	*l* = −8→9
30937 measured reflections	

### Refinement

**Table:**

Refinement on *F*^2^	Primary atom site location: structure-invariant direct methods
Least-squares matrix: full	Secondary atom site location: difference Fourier map
*R*[*F*^2^ > 2σ(*F*^2^)] = 0.032	Hydrogen site location: inferred from neighbouring sites
*wR*(*F*^2^) = 0.068	H atoms treated by a mixture of independent and constrained refinement
*S* = 0.85	*w* = 1/[σ^2^(*F*_o_^2^) + (0.0369*P*)^2^] where *P* = (*F*_o_^2^ + 2*F*_c_^2^)/3
2602 reflections	(Δ/σ)_max_ = 0.001
190 parameters	Δρ_max_ = 0.14 e Å^−^^3^
0 restraints	Δρ_min_ = −0.15 e Å^−^^3^

### Special details

**Table:**

Geometry. All e.s.d.'s (except the e.s.d. in the dihedral angle between two l.s. planes) are estimated using the full covariance matrix. The cell e.s.d.'s are taken into account individually in the estimation of e.s.d.'s in distances, angles and torsion angles; correlations between e.s.d.'s in cell parameters are only used when they are defined by crystal symmetry. An approximate (isotropic) treatment of cell e.s.d.'s is used for estimating e.s.d.'s involving l.s. planes.
Refinement. Refinement of *F*^2^ against ALL reflections. The weighted *R*-factor *wR* and goodness of fit *S* are based on *F*^2^, conventional *R*-factors *R* are based on *F*, with *F* set to zero for negative *F*^2^. The threshold expression of *F*^2^ > 2σ(*F*^2^) is used only for calculating *R*-factors(gt) *etc*. and is not relevant to the choice of reflections for refinement. *R*-factors based on *F*^2^ are statistically about twice as large as those based on *F*, and *R*- factors based on ALL data will be even larger.

### Fractional atomic coordinates and isotropic or equivalent isotropic displacement parameters (Å^2^)

**Table:**

	*x*	*y*	*z*	*U*_iso_*/*U*_eq_	
O1	0.36193 (5)	0.68130 (4)	0.21617 (11)	0.0266 (2)	
H1A	0.3139	0.6810	0.2513	0.040*	
N1	0.30745 (7)	0.68654 (6)	0.83127 (16)	0.0257 (3)	
C1	0.54376 (8)	0.63700 (5)	0.11634 (15)	0.0185 (3)	
C2	0.53840 (8)	0.57371 (6)	0.04034 (16)	0.0240 (3)	
H2A	0.5560	0.5434	0.1227	0.029*	
H2B	0.4813	0.5649	0.0102	0.029*	
C3	0.59193 (8)	0.56895 (6)	−0.11291 (17)	0.0271 (4)	
H3	0.5875	0.5274	−0.1599	0.033*	
C4	0.68033 (8)	0.58181 (6)	−0.06886 (18)	0.0290 (4)	
H4A	0.6998	0.5519	0.0127	0.035*	
H4B	0.7147	0.5785	−0.1688	0.035*	
C5	0.68711 (8)	0.64514 (6)	0.00313 (17)	0.0258 (3)	
H5	0.7450	0.6536	0.0332	0.031*	
C6	0.65776 (8)	0.69158 (6)	−0.12231 (18)	0.0281 (4)	
H6A	0.6923	0.6900	−0.2222	0.034*	
H6B	0.6619	0.7326	−0.0746	0.034*	
C7	0.56932 (8)	0.67818 (6)	−0.16850 (17)	0.0254 (3)	
H7	0.5503	0.7082	−0.2519	0.030*	
C8	0.51604 (8)	0.68229 (6)	−0.01404 (15)	0.0228 (3)	
H8A	0.5191	0.7236	0.0319	0.027*	
H8B	0.4587	0.6741	−0.0440	0.027*	
C9	0.56360 (8)	0.61470 (6)	−0.24141 (16)	0.0282 (4)	
H9A	0.5067	0.6060	−0.2737	0.034*	
H9B	0.5981	0.6118	−0.3412	0.034*	
C10	0.63360 (8)	0.64953 (6)	0.15736 (17)	0.0242 (3)	
H10A	0.6387	0.6904	0.2056	0.029*	
H10B	0.6527	0.6201	0.2405	0.029*	
C11	0.49559 (8)	0.64112 (6)	0.27851 (16)	0.0226 (3)	
H11A	0.5205	0.6125	0.3574	0.027*	
H11B	0.5039	0.6821	0.3238	0.027*	
C12	0.40426 (8)	0.62906 (6)	0.27706 (16)	0.0221 (3)	
H12	0.3932	0.5945	0.2009	0.027*	
C13	0.37566 (8)	0.61198 (6)	0.44839 (16)	0.0197 (3)	
C14	0.35838 (7)	0.65598 (6)	0.56444 (16)	0.0199 (3)	
H14	0.3645	0.6972	0.5356	0.024*	
C15	0.33224 (7)	0.64093 (6)	0.72247 (17)	0.0209 (3)	
C16	0.32485 (8)	0.58040 (6)	0.76474 (17)	0.0250 (3)	
H16	0.3071	0.5694	0.8721	0.030*	
C17	0.34335 (8)	0.53647 (6)	0.65039 (18)	0.0277 (4)	
H17	0.3390	0.4952	0.6804	0.033*	
C18	0.36811 (8)	0.55162 (6)	0.49282 (17)	0.0259 (3)	
H18	0.3800	0.5209	0.4149	0.031*	
H1B	0.3144 (9)	0.6768 (6)	0.942 (2)	0.045 (5)*	
H1C	0.3315 (8)	0.7235 (7)	0.8081 (17)	0.033 (4)*	

### Atomic displacement parameters (Å^2^)

**Table:**

	*U*^11^	*U*^22^	*U*^33^	*U*^12^	*U*^13^	*U*^23^
O1	0.0216 (5)	0.0341 (5)	0.0241 (6)	0.0052 (4)	0.0027 (5)	0.0062 (5)
N1	0.0280 (7)	0.0324 (8)	0.0166 (8)	0.0026 (6)	0.0010 (6)	0.0003 (6)
C1	0.0189 (8)	0.0208 (7)	0.0158 (8)	0.0003 (6)	0.0010 (6)	−0.0002 (6)
C2	0.0250 (8)	0.0223 (7)	0.0247 (8)	−0.0017 (6)	0.0019 (6)	0.0005 (6)
C3	0.0303 (8)	0.0224 (7)	0.0286 (9)	−0.0005 (6)	0.0046 (7)	−0.0073 (7)
C4	0.0272 (9)	0.0322 (8)	0.0276 (9)	0.0082 (6)	0.0086 (7)	0.0036 (7)
C5	0.0160 (7)	0.0359 (8)	0.0257 (9)	−0.0040 (6)	−0.0009 (6)	0.0016 (7)
C6	0.0296 (9)	0.0280 (8)	0.0268 (9)	−0.0056 (6)	0.0071 (7)	0.0018 (7)
C7	0.0270 (8)	0.0281 (8)	0.0210 (8)	0.0034 (6)	0.0015 (6)	0.0073 (7)
C8	0.0212 (7)	0.0241 (7)	0.0230 (8)	0.0027 (6)	−0.0003 (6)	0.0011 (6)
C9	0.0255 (8)	0.0403 (9)	0.0188 (8)	−0.0011 (7)	0.0026 (7)	−0.0045 (7)
C10	0.0239 (8)	0.0267 (8)	0.0221 (8)	0.0004 (6)	−0.0040 (7)	0.0005 (6)
C11	0.0252 (8)	0.0241 (7)	0.0185 (8)	−0.0008 (6)	−0.0023 (6)	0.0001 (6)
C12	0.0229 (8)	0.0232 (7)	0.0202 (8)	0.0023 (6)	0.0005 (7)	−0.0006 (6)
C13	0.0157 (7)	0.0256 (7)	0.0177 (8)	−0.0003 (6)	−0.0007 (6)	0.0008 (6)
C14	0.0174 (7)	0.0217 (7)	0.0206 (8)	−0.0004 (6)	−0.0006 (6)	0.0044 (6)
C15	0.0153 (7)	0.0291 (8)	0.0182 (8)	0.0010 (6)	−0.0018 (6)	0.0001 (6)
C16	0.0214 (8)	0.0331 (8)	0.0205 (9)	−0.0026 (6)	0.0009 (6)	0.0081 (7)
C17	0.0279 (9)	0.0239 (8)	0.0315 (10)	−0.0037 (6)	−0.0023 (7)	0.0070 (7)
C18	0.0277 (8)	0.0244 (7)	0.0255 (9)	0.0005 (6)	−0.0004 (7)	−0.0022 (7)

### Geometric parameters (Å, °)

**Table:**

O1—C12	1.4393 (14)	C7—C9	1.5302 (18)
O1—H1A	0.8400	C7—C8	1.5305 (17)
N1—C15	1.4027 (17)	C7—H7	1.0000
N1—H1C	0.930 (14)	C8—H8A	0.9900
N1—H1B	0.934 (16)	C8—H8B	0.9900
C1—C8	1.5277 (16)	C9—H9A	0.9900
C1—C2	1.5360 (16)	C9—H9B	0.9900
C1—C11	1.5373 (17)	C10—H10A	0.9900
C1—C10	1.5397 (18)	C10—H10B	0.9900
C2—C3	1.5258 (17)	C11—C12	1.5257 (18)
C2—H2A	0.9900	C11—H11A	0.9900
C2—H2B	0.9900	C11—H11B	0.9900
C3—C4	1.5240 (18)	C12—C13	1.5141 (17)
C3—C9	1.5271 (18)	C12—H12	1.0000
C3—H3	1.0000	C13—C14	1.3850 (17)
C4—C5	1.5254 (18)	C13—C18	1.3923 (17)
C4—H4A	0.9900	C14—C15	1.3915 (18)
C4—H4B	0.9900	C14—H14	0.9500
C5—C6	1.5258 (18)	C15—C16	1.3912 (17)
C5—C10	1.5317 (18)	C16—C17	1.3789 (19)
C5—H5	1.0000	C16—H16	0.9500
C6—C7	1.5311 (18)	C17—C18	1.3817 (18)
C6—H6A	0.9900	C17—H17	0.9500
C6—H6B	0.9900	C18—H18	0.9500
			
C12—O1—H1A	109.5	C1—C8—H8A	109.5
C15—N1—H1C	112.7 (9)	C7—C8—H8A	109.5
C15—N1—H1B	113.8 (9)	C1—C8—H8B	109.5
H1C—N1—H1B	110.3 (13)	C7—C8—H8B	109.5
C8—C1—C2	107.88 (10)	H8A—C8—H8B	108.1
C8—C1—C11	113.47 (10)	C3—C9—C7	109.26 (11)
C2—C1—C11	111.57 (10)	C3—C9—H9A	109.8
C8—C1—C10	108.48 (10)	C7—C9—H9A	109.8
C2—C1—C10	107.86 (10)	C3—C9—H9B	109.8
C11—C1—C10	107.42 (10)	C7—C9—H9B	109.8
C3—C2—C1	110.89 (10)	H9A—C9—H9B	108.3
C3—C2—H2A	109.5	C5—C10—C1	111.33 (11)
C1—C2—H2A	109.5	C5—C10—H10A	109.4
C3—C2—H2B	109.5	C1—C10—H10A	109.4
C1—C2—H2B	109.5	C5—C10—H10B	109.4
H2A—C2—H2B	108.1	C1—C10—H10B	109.4
C2—C3—C4	110.29 (11)	H10A—C10—H10B	108.0
C2—C3—C9	109.45 (11)	C12—C11—C1	119.36 (11)
C4—C3—C9	109.04 (11)	C12—C11—H11A	107.5
C2—C3—H3	109.3	C1—C11—H11A	107.5
C4—C3—H3	109.3	C12—C11—H11B	107.5
C9—C3—H3	109.3	C1—C11—H11B	107.5
C5—C4—C3	109.38 (11)	H11A—C11—H11B	107.0
C5—C4—H4A	109.8	O1—C12—C13	111.45 (10)
C3—C4—H4A	109.8	O1—C12—C11	109.72 (10)
C5—C4—H4B	109.8	C13—C12—C11	110.04 (11)
C3—C4—H4B	109.8	O1—C12—H12	108.5
H4A—C4—H4B	108.2	C13—C12—H12	108.5
C6—C5—C4	110.13 (11)	C11—C12—H12	108.5
C6—C5—C10	108.59 (11)	C14—C13—C18	118.96 (12)
C4—C5—C10	109.19 (11)	C14—C13—C12	120.66 (11)
C6—C5—H5	109.6	C18—C13—C12	120.37 (12)
C4—C5—H5	109.6	C13—C14—C15	121.27 (12)
C10—C5—H5	109.6	C13—C14—H14	119.4
C5—C6—C7	109.41 (11)	C15—C14—H14	119.4
C5—C6—H6A	109.8	C14—C15—C16	119.03 (12)
C7—C6—H6A	109.8	C14—C15—N1	119.67 (12)
C5—C6—H6B	109.8	C16—C15—N1	121.08 (13)
C7—C6—H6B	109.8	C17—C16—C15	119.84 (13)
H6A—C6—H6B	108.2	C17—C16—H16	120.1
C6—C7—C9	109.36 (11)	C15—C16—H16	120.1
C6—C7—C8	109.41 (11)	C16—C17—C18	120.94 (13)
C9—C7—C8	109.60 (10)	C16—C17—H17	119.5
C6—C7—H7	109.5	C18—C17—H17	119.5
C9—C7—H7	109.5	C17—C18—C13	119.95 (13)
C8—C7—H7	109.5	C17—C18—H18	120.0
C1—C8—C7	110.82 (10)	C13—C18—H18	120.0

### Hydrogen-bond geometry (Å, °)

**Table:**

*D*—H···*A*	*D*—H	H···*A*	*D*···*A*	*D*—H···*A*
O1—H1A···N1^i^	0.84	2.10	2.9400 (14)	176.
N1—H1C···O1^ii^	0.930 (15)	2.295 (15)	3.2048 (16)	166.0 (13)
N1—H1B···O1^iii^	0.930 (16)	2.357 (16)	3.2472 (16)	160.1 (14)

Symmetry codes: (i) −*x*+1/2, *y*, *z*−1/2; (ii) *x*, −*y*+3/2, *z*+1/2; (iii) *x*, *y*, *z*+1.
